# Non-pharmacological, multicomponent group therapy in patients with degenerative dementia: a 12-month randomzied, controlled trial

**DOI:** 10.1186/1741-7015-9-129

**Published:** 2011-12-01

**Authors:** Elmar Graessel, Renate Stemmer, Birgit Eichenseer, Sabine Pickel, Carolin Donath, Johannes Kornhuber, Katharina Luttenberger

**Affiliations:** 1 Friedrich-Alexander-Universität Erlangen-Nürnberg, Clinic for Psychiatry and Psychotherapy, Department of Medical Psychology and Medical Sociology, Schwabachanlage 6, 91054 Erlangen, Germany; 2 Catholic University of Applied Sciences Mainz, Department of Health and Nursing, Saarstrasse 3, 55122 Mainz, Germany

**Keywords:** dementia, non-pharmacological intervention, group therapy, RCT, nursing home

## Abstract

**Background:**

Currently available pharmacological and non-pharmacological treatments have shown only modest effects in slowing the progression of dementia. Our objective was to assess the impact of a long-term non-pharmacological group intervention on cognitive function in dementia patients and on their ability to carry out activities of daily living compared to a control group receiving the usual care.

**Methods:**

A randomized, controlled, single-blind longitudinal trial was conducted with 98 patients (follow-up: n = 61) with primary degenerative dementia in five nursing homes in Bavaria, Germany. The highly standardized intervention consisted of motor stimulation, practice in activities of daily living, and cognitive stimulation (acronym MAKS). It was conducted in groups of ten patients led by two therapists for 2 hours, 6 days a week for 12 months. Control patients received treatment as usual. Cognitive function was assessed using the cognitive subscale of the Alzheimer's Disease Assessment Scale (ADAS-Cog), and the ability to carry out activities of daily living using the Erlangen Test of Activities of Daily Living (E-ADL test) at baseline and after 12 months.

**Results:**

Of the 553 individuals screened, 119 (21.5%) were eligible and 98 (17.7%) were ultimately included in the study. At 12 months, the results of the per protocol analysis (n = 61) showed that cognitive function and the ability to carry out activities of daily living had remained stable in the intervention group but had decreased in the control patients (ADAS-Cog: adjusted mean difference: -7.7, 95% CI -14.0 to -1.4, *P *= 0.018, Cohen's d = 0.45; E-ADL test: adjusted mean difference: 3.6, 95% CI 0.7 to 6.4, *P *= 0.015, Cohen's d = 0.50). The effect sizes for the intervention were greater in the subgroup of patients (n = 50) with mild to moderate disease (ADAS-Cog: Cohen's d = 0.67; E-ADL test: Cohen's d = 0.69).

**Conclusions:**

A highly standardized, non-pharmacological, multicomponent group intervention conducted in a nursing-home setting was able to postpone a decline in cognitive function in dementia patients and in their ability to carry out activities of daily living for at least 12 months.

**Trial Registration:**

http://www.isrctn.com Identifier: ISRCTN87391496

## Background

In the absence of effective treatment for the causes of degenerative dementias, the primary objective of pharmacological and non-pharmacological therapy remains to slow disease progression. Although acetylcholinesterase inhibitors have been shown to have a positive impact on cognitive function in patients with Alzheimer's disease and on their ability to carry out activities of daily living (ADL) [1-3], these agents also have a variety of dose-dependent adverse effects [2-4]. These and the limited efficacy [3,5] of currently available anti-dementia drugs have led to increased scientific interest in non-pharmacological interventions.

A wide array of such interventions has been developed over the past two decades [6-9], ranging from cognitive training [10] and music therapy [11] to biographical approaches [12] and sensory stimulation [13,14]. Cognitive training, especially, has been evaluated in a number of randomized controlled trials (RCTs). In a recent randomized trial with small sample size, for example [15], there was a significant improvement in the ADAS-Cog after a six-month-cognitive intervention. Yet this effect could only be seen in the subgroup of patients with Mild Cognitive Impairment. In another recent RCT [16] Spector *et al*. also found a significant effect of a 14-session cognitive group treatment on the total ADAS-Cog (*P *= 0.01). Most of the different approaches mentioned above, however, have involved unimodal therapy and have demonstrated limited effectiveness [15,17], if they have been evaluated at all. It seems reasonable to assume that because people who live independently are confronted in their everyday lives with multiple challenges and stimuli, interventions aimed at slowing disease progression in dementia patients should also consist of multiple components [4]. This has been underscored by a recent systematic review, which demonstrated the efficacy of multicomponent interventions for dementia patients in achieving a range of outcomes [18]. In their review, the authors found a Grade B recommendation for multicomponent interventions for dementia patients for improvement in cognition and ADL. Of all 179 studies included, the authors detected only 13 high quality trials regarding different interventions (one for cognitive training, none for abilities of daily living). One multicomponent intervention combining cognitive and motor elements [19] had significant effects on cognitive abilities after 12 months but no significant effect on patients' abilities to carry out ADL. In another RCT combining reality orientation training with reminiscence therapy [20], the authors found a significant effect on cognition immediately after intervention. We thus designed a therapy known by the acronym MAKS, with each letter standing for a component of the intervention: M for motor stimulation, A for ADL, K for cognitive stimulation (the German word being *kognitiv*), and S for a short introductory phase with what we called a spiritual element (for example, discussing topics such as happiness or singing a song, usually a hymn). The cognitive component aimed to have a direct effect, and the motor exercises an indirect effect [7,21-23], on higher cognitive functions [24-26]. Practicing ADL was intended to slow the loss of independence typically seen in dementia patients [27,28].

Proving a causal relationship between a non-pharmacological intervention and a therapeutic effect requires a rigorous methodological approach, such as that demanded of pharmacological studies, including a randomized, controlled design, validated outcome measures, blinded testing, control for other medication and non-drug influences, and recording of serious adverse events [29]. The present investigation attempts to meet these standards. We hypothesized that patients who participated in the MAKS intervention would show better cognitive function and less impairment in ADL than control patients after 12 months. The results for the other measures used in the study-direct care time, neuropsychiatric symptoms, and functional independence-will be reported elsewhere.

## Methods

### Study design

**Figure 1:**
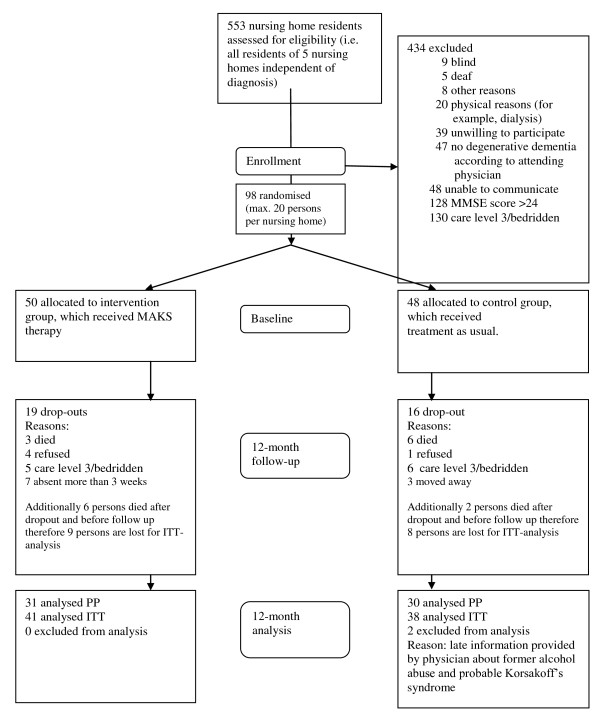
**CONSORT flowchart of study design**.

We conducted a randomized, controlled, single-blind longitudinal study examining the efficacy of a multicomponent, non-pharmacological group therapy known as MAKS in dementia patients in five German nursing homes. Therapy was conducted six days a week and lasted for twelve months, beginning in December 2008 and ending in December 2009 (Figure 1).

We determined the size of the sample based on the interim results of a pilot study [30], which used the same outcome measures as in the present analysis and showed an effect size of 0.67 (Cohen's d) for ADL in an experimental-control group comparison after six months. Power analysis (one-tailed testing) revealed that a sample size of 29 in each group (that is, 58 patients in total) would be required, with the significance level (α) and test power (1-β) set at 0.05 and 0.80, respectively.

Between October and December 2008, we screened all residents (N = 553) in five nursing homes in Mittelfranken, a district within the German state of Bavaria. The nursing homes were of a similar size (between 100 and 134 residents) and were all operated by the same organization, the Diakonie Neuendettelsau. Therefore, they were also similar in terms of corporate philosophy and the range of non-MAKS activities offered by the homes. We applied the following inclusion criteria: presence of primary degenerative dementia according to ICD-10 (F00, F03, or G30) and as confirmed by the attending physician; fewer than 24 points on the Mini-Mental State Examination (MMSE) [31]; and written informed consent by patients and, where necessary, their legal guardians prior to baseline. The form sheet, all legal conditions and the study design were examined by the Ethics Committee of the Medical Faculty of the University of Erlangen. Approval was granted on 10 July 2008 (Registration Number 3232). Exclusion criteria were as follows: vascular (F01) or secondary (F02) dementia according to ICD-10; the presence of other neurological/psychiatric diseases that could explain patients' decline in cognitive function (such as addiction, major depression, or schizophrenia); very high nursing care needs (that is, care level 3, which is the highest level on the three-level scale currently used to determine eligibility for nursing care benefits in Germany); deafness; or blindness. Taking medication of any type did not affect inclusion or exclusion in our study (see Table 1 for medication taken).

**Table 1:** Characteristics of patients (randomized at baseline, n = 96)

Characteristics	Intervention group (n = 50)	Control group (n = 46)	Total (n = 96)	Test for group differences			Test for nursing home differences^f^	
				χ^2^	t^e ^	*P *Value	χ^2 ^	*P *Value
**Age, mean (SD)**	84.5 (4.5)	85.7 (5.7)	85.1 (5.1)		1.19	0.24	4.91	0.30
**Women, no. (%)**	44 (88.0)	36 (78.3)	80 (83.3)	1.64		0.20	5.84	0.21
**Educational attainment, no. (%)**				3.85		0.43	14.08	0.01
No school completed	5 (10.9)	9 (20.9)	14 (15.7)					
Elementary/secondary school	39 (84.7)	30 (69.8)	69 (77.5)					
University preparatory	2 (4.3)	3 (7.0)	5 (5.6)					
University	0 (0.0)	1 (2.3)	1 (1.1)					
**Marital status, no. (%)**				5.25		0.15	2.62	0.62
Married	4 (8.0)	9 (19.6)	13 (13.5)					
Widowed	40 (80.0)	34 (73.9)	74 (77.1)					
Divorced	0 (0)	1 (2.2)	1 (1)					
Single	6 (12.0)	2 (4.3)	8 (8.3)					
**MMSE mean (SD)**	15.4 (5.4)	13.8 (5.4)	14.6 (5.4)		-1.45	0.15	1.68	0.79
**NOSGER subscale mood, mean (SD)**	10.6 (3.1)	9.9 (3.0)	10.3 (3.1)		-1.07	0.29	5.03	0.29
**Care level^a^, no. (%)**				4.63		0.10	4.56	0.34
None	7 (14.0)	2 (4.3)	9 (9.4)					
1	27 (54.0)	21 (45.7)	48 (50.0)					
2	16 (32.0)	23 (50.0)	39 (40.6)					
3	0 (0)	0 (0)	0 (0)					
**Charlson comorbidity index^b^, mean (SD)**	1.1 (1.6)	1.1 (1.6)	1.1 (1.4)		-0.31	0.76	7.35	0.12
**Use of anti-dementia med.^c^, no. (%)**	9 (18.0)	4 (8.7)	13 (13.5)	1.80		0.18	5.48	0.24
**Medication score^d^, mean (SD)**	-1.4 (1.7)	-1.5 (1.7)	-1.5 (1.7)		-0.11	0.91	0.93	0.92
**ADAS-Cog, mean (SD)**	33.5 (13.1)	38.0 (14.4)	35.6 (13.8)		1.60	0.11	12.11	0.02
**E-ADL test, mean (SD)**	25.9 (5.4)	23.7 (5.9)	24.7 (5.7)		-1.62	0.10	9.69	0.05

Abbreviations: MMSE, Mini-Mental State Examination; NOSGER, Nurses' Observation Scale for Geriatric Patients; ADAS-Cog, Alzheimer's Disease Assessment Scale, subscale cognition; E-ADL test, Erlangen Test of Activities of Daily Living.

^a^The care level describes the extent to which nursing care is needed: none (no needs), 1 (moderate needs), 2 (high needs), and 3 (very high needs). This scale is used in Germany to establish eligibility for nursing care benefits. Put simply, individuals who need regular assistance with activities of everyday living and household chores for a daily average of at least 1.5 hours are assigned to level 1, those who need at least 3 hours of assistance to level 2, and those who need 5 or more hours of assistance to level 3. The classification is conducted by an organization formed by the long-term care insurance funds, which are statutory entities that administer the system of long-term care insurance in Germany. ^b^Charlson comorbidity index: Effect of comorbidities (that is, in addition to dementia) on mortality rate. A condition is assigned a score according to the mortality risk associated with it. One-year mortality increases from 12% (index = 0) to 85% (index ≥ 5) as the score increases. ^c^Anti-dementia medication during the intervention period: intervention group: 3× acetylcholinesterase (AChE) inhibitors, 7× memantine, 1× both; control group: 2× AChE inhibitors, 2× memantine. ^d^Medication score: mean value of all medications taken by the patient. It was calculated using all prescribed medications at baseline, including anti-dementia drugs. To do so, two experts from the University of Erlangen's Department of Clinical Pharmacology rated all medications in terms of their sedating or stimulating effect or side effect using a 5-step scale: -2 (very sedating), -1 (sedating), 0 (neither sedating nor stimulating), +1 (stimulating), +2 (very stimulating). **^e^**df = 94. **^f^**with Kruskal-Wallis or Pearson χ^2 ^with df = 5.

Of the 119 patients determined to be eligible (that is, between 18 and 27 patients in each of the 5 nursing homes), 98 were ultimately randomized to either the MAKS or the control group one week prior to the commencement of therapy. We computer generated a randomization list for each home, assigning 10 patients to each treatment group and 10 patients to each control group. In one nursing home, only 18 eligible patients could be randomized (10 to the treatment group and 8 to the control group). Control patients received treatment as usual instead of MAKS therapy.

The study did not interfere in any way with patients' existing pharmacological treatment, nursing care, or with their participation in the regular activities offered by the nursing home outside of the MAKS study. Independent evaluators, who were blinded to treatment allocation and were not part of the nursing home staff, recorded the outcome measures at baseline (before commencement of therapy) and after 12 months (in December 2009/January 2010, after completion of therapy). Data were anonymized and submitted to the central study site.

### Quality assurance measures

The evaluators took part in two training sessions with actor patients prior to the start of data recording and in a third training session six months later. The nursing home staff, which included therapists, aides, and one study coordinator per nursing home, also received training in data recording. To ensure the quality of the data recorded at the five participating nursing homes, the study design was explained in detail to the coordinators. Assistants from the central study site conducted random on-location checks of the coordinators' documentation. In a manner analogous to that used in drug studies, nursing home staff recorded and reported any serious adverse events that occurred during the study period. These were defined as falls resulting in injury, serious injury of any other type requiring a physician's attention, or death. A Data Monitoring and Safety Board, which consisted of four external experts from different professions, oversaw the implementation of the study and monitored for serious adverse events every three months.

### Implementation of treatment

MAKS therapy was conducted in each nursing home by two therapists and one aide from Monday through Saturday from 9:30 am to 11:30 am for 12 months. The therapists were registered nurses for the elderly. Each therapy group consisted of 10 dementia patients. Therapists and aides received a standardized handbook from the central study site describing in detail the steps to be taken on each day of therapy. This guaranteed that on any given day the same tasks would be performed at each nursing home. The handbook was developed specifically for the present study by a study scientist (psychologist).

To ensure that the handbook was used in a standardized manner in all five nursing homes, we provided therapists and aides with two complete days of training prior to the commencement of therapy and one day of follow-up training after four months. A team from the central study site monitored compliance with the handbook by conducting three on-location checks per home. As an additional quality assurance measure, therapists were required to record any deviations from the handbook. Compliance with the handbook was 97.5% in the five nursing homes.

Attendance of participants was stringently monitored by therapists and study coordinators in the homes. Participants who completed the whole intervention period only missed 3% of the intervention days on average. The minimum attendance for not being excluded from the study was 50%.

### Treatment conditions

MAKS is a multicomponent group therapy consisting of tasks organized into three categories-motor stimulation (M), ADL (A), and cognition (K)-preceded by a short introduction consisting of what we called a spiritual element (S).

Each daily session began with this introduction, which lasted approximately 10 minutes and was designed to help the dementia patients feel part of the group. It consisted of a round of greetings followed by a group song (usually a hymn) or a discussion about a meaningful topic, such as happiness. This was followed by about 30 minutes of motor exercises, such as bowling, croquet, or balancing a tennis ball on a frisbee and passing it to one's neighbor. After a 10 minute break, the patients spent approximately 30 minutes completing a variety of cognitive tasks, ranging from paper and pencil exercises, such as solving word jumbles or matching symbols into pairs, to picture puzzles projected digitally onto a large screen to be solved by the group. MAKS was designed to promote activity that takes place at an individual's performance limit. Therefore, therapists matched all participants into three homogenous groups according to the individual performance levels (operationalized with MMSE-score) and assigned the cognitive tasks from one of three difficulty levels to the appropriate group. This was followed by about 40 minutes during which patients carried out ADL (such as preparing a snack), engaged in creative tasks (such as working with wood, paper, or other natural materials), or did simple gardening work (for further examples see Appendix 2 and 3 in Additional file 1).

The control group received the usual care offered in each nursing home and were free to participate in any of the regular, non-MAKS activities offered at the home, such as memory training, physical exercises to reduce the risk of falling, cooking groups, or occupational therapy (for an overview of all non-MAKS activities see Appendix 4 in Additional file 1). Patients in the control group participated in an average of two of these non-MAKS activities per week. Patients in the MAKS group were also free to take part in these activities in addition to MAKS and did so once a week on the average.

### Outcome measures

The two outcome measures included in the present analysis-cognitive function and the ability to carry out ADL-were recorded by evaluators (students of Psychology in their last year) who had received training, did not belong to the nursing home staff, and were blinded to treatment allocation. During the examination, which took place in each patient's room, only the patient, the evaluator, and, if necessary, a nurse were present. The evaluator used the cognitive subscale of the Alzheimer's Disease Assessment Scale (ADAS-Cog) [32] to measure function. The scoring range for ADAS-Cog is from 0 to 70, with higher scores indicating greater cognitive impairment (Cronbach's α = 0.82; correlation with MMSE -0.81) [32]. In addition, the evaluator used the Erlangen Test of ADL (E-ADL test) [33] to measure the ability of dementia patients to carry out basic ADLs under standardized conditions (that is, pouring a drink, cutting a piece of bread, opening a small cupboard, washing one's hands, and tying a bow). The scoring range for the E-ADL test is 0 to 30, with higher scores indicating less impairment (α = 0.77). The E-ADL test was originally validated in nursing homes (correlation with the Nurses' Observation Scale for Geriatric Patients (NOSGER): -0.60) [33].

### Other measures

Study coordinators recorded each patient's age, gender, educational attainment, family status, and nursing care needs at baseline. Nursing staff rated depressive symptoms among the patients at baseline using the mood subscale of NOSGER (NOSGER-mood; test-retest reliability: 0.85; correlation with the Geriatric Depression Scale: r_S _= 0.63). Nursing care needs were determined based on the three-level scale used in Germany to establish eligibility for nursing care benefits. The scale ranges from care level 1 (moderate needs) to care level 3 (very high needs), and each potential nursing home resident in Germany is evaluated by the so-called long-term care funds (*Pflegekassen*), which are quasi-public entities that administer the German system of statutory long-term care insurance.

We also calculated the effect of any previous medical diagnoses on the mortality rate using the Charlson comorbidity index [34]. Potential bias resulting from non-pharmacological and pharmacological interventions was accounted for by using a participation score (regular, non-MAKS activities offered by the nursing homes during the time of intervention) and a medication score (sedative/stimulating effect of all medication at baseline). Changes in prescription during the intervention time were also recorded but occurred only in 16% of all cases and were therefore not taken into account (for further information see Tables 1 and 2).

### Patients

All patients included in the study were white. At baseline, there were no statistically significant differences between the control and intervention groups (Table 1). Only 13 patients (13.5%) were taking anti-dementia medication at baseline. The patients who were excluded (n = 434; for reasons see Figure 1) were an average of 83.1 years old and 77% were women, making them similar to the study participants in terms of age and gender (see Table 1). Of the 98 patients ultimately included in the study, 35 fulfilled our dropout criteria during the 12-month intervention (death, being bedridden, care level 3, moving away, less than 50% participation, 3 weeks or more in hospital, refusal of therapy or study participation): 16 in the control group and 19 in the intervention group. Distribution of dropouts was between 2 and 4 in all months with the only exceptions being months 7 and 10 without any dropouts and month 12 with 7 dropouts. Additionally, two patients had to be excluded because they had received an incorrect diagnosis (see Figure 1). There were no differences between the nursing homes with respect to participants' age, gender, family status, MMSE score, use of anti-dementia medication, or Charlson index scores (see Table 1). There were, however, significant differences between the nursing homes in terms of patients' educational attainment and baseline scores on the E-ADL test and ADAS-Cog subscale. We adjusted for these differences in our regression analysis.

### Statistical analysis

We planned the study and assessed its results in collaboration with the Institute for Medical Informatics, Biometry, and Epidemiology at the University of Erlangen, Germany. In accordance with the study protocol, we tested the study hypothesis in a per protocol (PP) analysis (that is, of patients who did not drop out). We also performed a sensitivity analysis according to an intention to treat (ITT) approach, which included all patients who had been randomly assigned to the two groups at baseline and were still alive after 12 months. In general, we attempted to record outcome variables for all patients who were still alive after 12 months (n = 79). If, however, more than 20% of the items on the ADAS-Cog subscale or the E-ADL test were missing for any given patient (for example, because of his or her refusal to complete the test), we computed the score according to the expectation maximum (EM) algorithm, with the variables showing the greatest proportion of explained variance in the missing variable. These were always the pertinent baseline value and the group assignment. The scores recorded for patients who died during the study period were not imputed. Under these conditions, imputation was necessary in 10% of cases in the ITT analysis and in no case in the PP analysis.

We examined group differences between the nursing homes using the Kruskal-Wallis test or the χ^2 ^test. Differences at baseline were examined either with the χ^2 ^test or with the *t *test for independent samples (Table 1). First, we tested differences between baseline and endpoint for each group separately using the *t *test for dependent samples (ADAS-Cog) and the Wilcoxon signed rank test for non-parametric data (E-ADL). We reported adjusted mean differences in the outcome variables with 95% confidence intervals. Moreover, we calculated effect sizes (Cohen's d) using pooled standard deviation [35]. We defined a treatment benefit as a stabilization of, or an improvement in, the respective outcome measure; this definition served as the basis for calculating the number needed to treat for each measure. Subsequently, we conducted multiple linear regression analyses using the 12-month follow-up scores for the ADAS-Cog subscale and the E-ADL test as dependent variables. The independent variables were included in the regression equation by the enter method. According to Altman [36], the number of independent variables used should not exceed the square root of the sample size (here: n = 61). In addition to the baseline values for each outcome variable and the group variable (MAKS versus control group), we thus included only those variables that showed sufficient variance (at least 10% per group in a dichotomous variable) in the sample or that were not in multicollinear relationship (r > .5) to the baseline values of the outcome variables (Table 2). This resulted in a regression model with eight independent variables that fulfilled the Altman criterion. We conducted our statistical analysis using PASW 18.0 with the significance level set at *P *< 0.05. All analyses were two-sided.

As an exploratory analysis Cohen's d for the matched subgroup of patients with only mild or moderate AD according to their initial MMSE Scores (mild (18 to 23), moderate (10 to 17) and severe (< 10) AD) was calculated.

As a sensitivity analysis in case of differences between nursing homes we performed a mixed model regression analysis with 'nursing home' as random effect, and group as allocation, along with a number of adjustment factors (gender, age, use of anti-dementia drugs, medication score, mood, participation in non-MAKS activities, baseline value) as fixed effects.

## Results

At baseline, there were no significant differences between patients who ultimately dropped out of the study (n = 35) and patients who completed the 12-month protocol (n = 61) in terms of age (dropouts mean: 85.5 years; completers mean: 84.9 years; *P *= 0.58), gender (dropouts 86% women; completers 82% women; *P *= 0.64), MMSE score (dropouts mean: 13.8; completers mean: 15.1: *P *= 0.27), or care level (dropouts/completers: none: 3%/13%; care level 1: 51%/49%; care level 2: 45%/38%; *P *= 0.24).

At the 12-month follow-up, the scores for the outcome measures in the intervention group remained unchanged as tested using the *t *test for dependent samples in the case of the ADAS-Cog subscale (baseline mean: 32.6 SD: 11.5; 12-month follow-up mean: 32.5 SD 15.3; *P *= 0.99) and using the Wilcoxon signed rank test in the case of the E-ADL test (baseline: 26.6 SD 5.1; 12-months: 26.3 SD 5.4; *P *= 0.71). In contrast, the control group showed an increase in impairment for both measures (that is, an increase in the ADAS-Cog score (baseline: 35.6 SD: 14.8; 12-months: 40.8 SD17.0; *P *= 0.039) and a decrease in the E-ADL test score (baseline: 24.3 SD: 5.6; 12-months: 21.5 SD: 7.4; *P *= 0.002). The number needed to treat (NNT) was 4.0 for the ADAS-Cog subscale and 5.5 for the E-ADL test. The adjusted mean differences between the two groups in the primary PP analysis (n = 61) were -7.7 points (95% CI -14.0 to -1.4; *P *= 0.018) for the ADAS-Cog subscale and 3.6 points (95% CI 0.7 to 6.4; *P *= 0.014) for the E-ADL test.

Multivariate regression analysis showed that participation in MAKS was a significant predictor of cognitive function and the ability to carry out ADL after 12 months (Table 2). Both regression models were significant at *P *< 0.001. In both regression equations, the baseline scores for the two outcome measures were significant predictors of the corresponding follow-up score. The number of additional, non-MAKS activities in which a patient took part was another significant predictor of the ADAS-Cog score (*P *= 0.03) after 12 months, with a higher number of activities being predictive of less cognitive impairment. Age, gender, NOSGER-mood score, the use of anti-dementia medication, and the impact of medication on psychomotor activities (medication score) had no predictive power.

**Table 2:** Multiple regression analysis with ADAS-Cog and E-ADL test as dependant variable (PP analysis, n = 61)

	ADAS-Cog (12-month follow-up)			E-ADL test (12-month follow-up)		
Independent variable^a^	Unstandardized β (95% CI)	*t*	*P *value	Unstandardized β (95% CI)	*t*	*P *value
Score^b ^at baseline	0.82 (0.59-1.05)	7.14	**< 0.001**	0.80 (0.54-1.06)	6.23	**< 0.001**
Group (control = 0 vs. MAKS = 1)	-7.67 (-13.97--1.37)	-2.44	**0.018**	3.57 (0.72-6.42)	2.52	**0.015**
Age	0.13 (-0.47-0.73)	0.43	0.67	0.08 (-0.19-0.35)	0.58	0.57
Gender	1.02 (-6.90-8.94)	0.26	0.80	0.91 (-2.64-4.46)	0.51	0.61
Medication score^c^	-0.38 (-2.14-1.39)	-0.43	0.67	-0.02 (-0.81-0.77)	-0.05	0.96
NOSGER, mood^d^	0.80 (-0.23-1.82)	1.56	0.13	-0.14 (-0.59-0.31)	-0.63	0.53
Participation score^e^	-0.07 (-0.14--0.01)	-2.22	**0.03**	0.01 (-0.02-0.04)	0.82	0.42
Use of anti-dementia medication^f^	-5.96 (-16.42-4.51)	-1.14	0.26	0.76 (-3.91-5.43)	0.33	0.74

P-values < 0.05 are in bold

Abbreviations: ADAS-Cog, Alzheimer's Disease Assessment Scale, subscale cognition; E-ADL test, Erlangen Test of Activities of Daily Living; PP, per protocol; NOSGER, Nurses' Observation Scale of Geriatric Patients.

^a^Excluded due to a lack of variance: educational attainment (93% with no more than elementary school education), excluded due to multicollinearity (r ≥ 0.50 with ADAS-Cog at baseline and/or E-ADL test at baseline): care level (0.76 with ADAS-Cog; 0.56 with the E-ADL test), Charlson comorbidity index (0.52 with ADAS-Cog); Mini Mental State Examination (0.71 with ADAS-Cog; 0.56 with the E-ADL test). ^b^ADAS-Cog at baseline if ADAS-Cog 12-month follow-up is dependent variable; E-ADL test at baseline if E-ADL test 12-month follow-up is dependent variable.

^c^Sedating (< 0) or stimulating (> 0) effect or side effect of the prescribed medications. ^d^NOSGER-mood subscale, which consists of five depression items rated on a scale of 1 (always) to 5 (never). The score ranges from 5 (no disturbance) to 25 (maximum possible degree of disturbance).

^e^Sum score of all non-MAKS activities regularly offered by the nursing homes and in which patients participated on a voluntary basis. The scores were recorded by nursing staff each week throughout the observation period. Examples of such activities are singing groups or physical exercises to reduce the risk of falling. Each activity in which a patient participated was rated with one point. ^f^Use of acetylcholinesterase (AChE) inhibitors or memantine.

In the PP analysis (n = 61), the effect size of MAKS therapy was moderate both for cognition (d = 0.45) and for the ability to perform ADL (d = 0.50). In the ITT analysis (n = 79), the effect sizes were d = 0.33 and d = 0.23, respectively. Looking at patients with mild to moderate dementia (MMSE 10 to 23) separately, the effect sizes increased to d = 0.67 for the ADAS-Cog subscale and d = 0.69 for the E-ADL test in the PP analysis (n = 50) and to d = 0.50 for the ADAS-Cog subscale and to d = 0.35 for the E-ADL test in the ITT analysis (n = 63).

Mixed model regression analysis yielded evidence of a significant impact of group allocation on ADAS-Cog (*P *= 0,008) and E-ADL test (*P *= 0,008), adjusted for the effect of nursing home allocation and a number of other adjustment factors.

A total of 67 serious adverse events occurred in the sample (n = 96) over 12 months, including 43 falls resulting in injury (MAKS: 19; control: 24), 7 other types of serious injury (MAKS: 3; control: 4), and 17 deaths (MAKS: 9; control: 8).

## Discussion

MAKS therapy had a significant effect on cognitive function in dementia patients and on their ability to carry out ADL in five participating nursing homes after 12 months compared to a control group that received usual care. The MAKS therapy was found to be an easily manageable form of therapy which was well-accepted by the patients. This is reflected in the very low number of missing days. Treatment with cholinesterase inhibitors or memantine, which was neither an inclusion nor an exclusion criterion in our study, had been prescribed to only 13 patients (13.5%; see Table 1) and perhaps had, therefore, no predictive power. The effect sizes of MAKS therapy were in the same range as those that have been reported for cholinesterase inhibitors with respect to cognition and about twice as high as those reported for cholinesterase inhibitors with respect to ADL [4]. One fourth of the patients in the intervention group dropped out during the study period or were lost to follow up, leading to lower effect sizes in our ITT analysis. In the intervention group, the effect sizes in patients with mild to moderate dementia were substantially higher than in patients with severe dementia. As a consequence, MAKS therapy, until further investigation with a larger sample size, should not be used in the latter patient group.

In a systematic search of the literature for comparable multicomponent, non-pharmacological RCTs in which the primary target group consisted of dementia patients and not of their caregiving family members, we were unable to identify any methodologically rigorous studies that had been conducted in a nursing home setting. Our search did reveal, however, that various combinations of non-pharmacological interventions have been conducted in community-dwelling dementia patients to date. Onor *et al*. [37], for example, investigated the efficacy of combined cognitive and occupational therapy but were unable, perhaps due to the small size of their sample (n = 32), to find any significant effects for the outcome measures cognition, ADL, or instrumental ADL. In another study, a significant effect on the ADAS-Cog subscale was demonstrated for an intensive intervention with 103 sessions that took place over a 12-month period and combined cognitive and motor elements in a sample similar in size (n = 84) to ours [19]. The evaluators were blinded to treatment allocation. Moreover, all patients had been receiving treatment with an acetylcholinesterase inhibitor and continued to do so during the study period. The authors, however, did not report effect sizes and only cursorily described the effects of the intervention at 12 months. A significant effect on patients' ability to carry out ADL could not be demonstrated. Gitlin et al. [38] investigated the efficacy of a multicomponent non-pharmacological intervention regarding functional abilities of community-dwelling dementia patients and several other outcomes. The intervention group (n = 102 dyads of dementia patient and caregiver) received up to 10 sessions with occupational therapists aimed at reducing environmental stressors and enhancing caregiver skills. Therapists identified strengths and deficits of the patients and trained caregivers in modifying home environment and communication. Additionally, health related information was assessed and recommendations were provided to the caregiver to share with the patients' physicians. The control dyads (n = 107) received up to three telephone calls from research staff and informational material was provided. After four months, significant improvements in functional dependence could be found for the intervention group compared to the control group. Cohen's d was 0.21, mainly due to an improvement in IADL-abilities. The effect could no longer be found after nine months. Outcome measures were caregiver ratings, obtained by interviewers blinded to participant group. In summary, our literature search revealed that the multicomponent interventions conducted among community-dwelling patients to date have shown only moderate effects. While this may be attributable to the type or intensity of therapy, it may also be related to the lack of consistently applied blinded performance tests. Because there was no evidence that the interventions in any of the identified studies had sustained effects, we defined the PP analysis as our primary evaluation strategy.

The present study has several important limitations. The first of these is the size of its sample, which consisted of 61 dementia patients in the PP analysis. This is in the middle range compared to the study samples in other non-pharmacological, multicomponent RCTs in dementia patients, which have had between 32 [37] and 209 [38] participants, albeit in a community-dwelling rather than a nursing-home setting. Future studies of MAKS therapy should include a larger sample of patients. The second limitation of our study is its lack of a control group receiving placebo treatment. In light of the many drawbacks of non-pharmacological placebo treatments, however, we feel that the use in our study of a control group receiving usual care was appropriate, especially considering that we placed no restrictions on patients in either group with regard to their continuation or initiation of any pharmacological or non-pharmacological treatment during the study period. All control group participants were allowed, for example, to continue to take part in the regular, non-MAKS activities offered by their nursing home, which they did on the average twice a week. As participation in these activities was voluntary, the significant effect on cognition after 12 months might either be due to helpfulness of these activities but also due to a convenience sample.

The treatment effects of MAKS therapy are undoubtedly attributable not only to specific but also to non-specific factors, such as the attention paid to patients by the therapists. To date, however, no studies have been able to demonstrate that paying more attention to patients, as for example is practiced in validation therapy, can produce a significant cognitive improvement in and of itself [39]. It therefore seems unlikely that the effects of MAKS therapy are attributable only to the greater intensity and duration of the attention paid to patients in the intervention group.

Our study also has a number of strengths compared to previous investigations. With only a few exceptions (for example, our decision not to include bed-ridden patients), our choice of inclusion and exclusion criteria closely reflects the clinical reality of dementia patients in nursing homes: Unlike many other studies of non-pharmacological interventions in this patient group, we did not exclude patients who showed poor cognitive function as measured by the MMSE or possible neuropsychiatric symptoms, such as challenging behavior. Another strength of our study was the rigorous standardization of MAKS therapy through the use of a handbook, enabling a high degree of agreement across the participating nursing homes. In addition to repeated trainings of the therapists, additional quality assurance measures were performed with all of the study and nursing home staff who participated in the investigation. Finally, in terms of methodology, another strength of our study was its use of external evaluators who were blinded to treatment allocation to assess both outcome variables. Moreover, additional factors that might influence treatment, such as medication or participation in additional, non-MAKS activities, were included in the multivariate analysis, and any serious adverse events were recorded.

Patients with dementia always experience limitations with respect to an age-appropriate 'participation in life' due to the symptoms of their disease and especially as nursing home residents. These limitations particularly affect social interaction, communication, cognition and everyday practical stimulation. The MAKS therapy aims at restoring this participation appropriate to the resources still available to the patient, and thus is characterized by multimodality, regularity and various degrees of difficulty.

Due to the standardization of the MAKS therapy and publication of a handbook [40], the therapy is easy to implement and requires little preparation time from the therapists. In the present study, the MAKS therapy was carried out in an intensive form. Performance as group therapy reduces the per-person costs of intervention. With two therapists for ten patients, the therapy costs are below €10/day and person and are thus still in the range of costs of therapy with acetylcholinesterase inhibitors. Moderately higher costs appear justified when the non-drug therapy shows no adverse effects and is at least as effective as therapy with acetylcholinesterase inhibitors. In the future, health economic studies to compare these costs to the possible savings which could result from stabilization of the patient's capabilities, and thus on the costs of care would be desirable.

The German health system includes, for example, so-called supplementary care services for the care of patients with dementia. The aim should be to use these resources in such a way that the dementia patient receives the maximum benefit. Depending on the health system, there are various types of resources which support dementia patients or aid in integrating them in everyday living. It appears promising to make appropriate use of the possibilities of non-drug therapy in the care of dementia patients.

## Conclusions

MAKS therapy preserved the cognitive function and ability to carry out ADL and thus, the independence, of dementia patients with mild to moderate dementia in nursing homes for at least 12 months without adverse effects. Future studies of MAKS therapy should include a larger sample of patients, more measurement points to see where benefits begin to occur and a longer follow-up period to determine if the stabilization seen at 12 months can be maintained for longer periods, and if there is any durable effect after treatment is stopped. It will also be important to determine whether MAKS therapy leads to improvements in standardized measures of quality of life.

Finally, future research should focus on any cumulative effects with anti-dementia drugs, and on the question of whether some form of MAKS therapy might be beneficial to dementia patients in an outpatient setting.

## Competing interests

The authors declare that they have no competing interests.

Research reported was funded by the German Ministry of Health (LT-Demenz-44-059)

Role of the sponsor: The German Ministry of Health had no role in designing or conducting the study; in collecting, analyzing, or interpreting the data; or in preparing, reviewing, or approving the manuscript. Researchers acted fully independent of the sponsor.

## Authors' contributions

All authors had full access to all of the data in the study. Study concept and design: EG, CD, JK, RS. Acquisition of data: EG, KL, BE. Analysis and interpretation of data: EG, KL, CD, SP, JK. Critical revision of the manuscript for important intellectual content: All authors. Statistical analyses: EG, KL, SP. Obtained funding: EG, RS, CD. Administrative, technical, or material support: RS, BE, EG, JK.

## Pre-publication history

The pre-publication history for this paper can be accessed here:

http://www.biomedcentral.com/1741-7015/9/129/prepub

## Supplementary Material

Additional file 1 **Appendices**. Click here for file
