# Supplementary material for: Non-pharmacological, multicomponent group therapy in patients with degenerative dementia: a 12-month randomzied, controlled trial
Source: BMC Med. 2011 Dec 1;9:129. doi: 10.1186/1741-7015-9-129 (PMC3254071; doi:10.1186/1741-7015-9-129)
Supplement: Additional file 1 — Appendices. [file 1741-7015-9-129-S1.DOC]

**APPENDICES**

**APPENDIX 1 Sensitivity analyses**

**APPENDIX 1 a. Multiple regression analysis with ADAS-Cog and E-ADL test as dependant variable (PP analysis, n=61)** **and with “care level” as additional independent variable**

|  | **ADAS-Cog (12-month follow-up)** | | | **E-ADL test (12-month follow-up)** | | |
| --- | --- | --- | --- | --- | --- | --- |
| **Independent variable^a^** | Unstandardised β (95% CI) | *t* | P value | Unstandardised β (95% CI) | *t* | P value |
| **Score^b^ at baseline** | 0.75 (0.48-1.03) | 5.43 | <**0.001** | 0.71 (0.42-1.00) | 4.95 | <**0.001** |
| **Group (control=0 vs. MAKS=1)** | -7.09 (-13.55—0.63) | -2.20 | **0.03** | 3.23 (0.36-6.11) | 2.26 | **0.03** |
| **Age** | 0.16 (0.45-0.76) | 0.52 | 0.61 | 0.04 (-0.23-0.32) | 0.33 | 0.75 |
| **Gender** | 0.37 (-7.72-8.45) | 0.09 | 0.93 | 1.26 (-2.31-4.83) | 0.71 | 0.48 |
| **Medication score^c^** | -0.38 (-2.15-1.39) | -0.43 | 0.67 | 0.02 (-0.77-0.81) | 0.06 | 0.95 |
| **NOSGER, mood^d^** | 0.82 (-0.21-1.85) | 1.59 | 0.12 | -0.12 (-0.57-0.33) | -0.54 | 0.60 |
| **Participation score^e^** | -0.08 (-0.14—0.01) | -2.24 | **0.03** | 0.01 (-0.02-0.04) | 0.94 | 0.35 |
| **Use of antidementia medication^f^** | -4.93 (-15.69-5.84) | -0.92 | 0.36 | 0.43 (-4.24-5.09) | 0.18 | 0.86 |
| **Care level^g^** | 2.43 (-3.21-8.07) | 0.87 | 0.39 | -1.53 (-3.87-0.82) | -1.31 | 0.20 |

Abbreviations: ADAS-Cog=Alzheimer’s Disease Assessment Scale, subscale cognition; E-ADL test=Erlangen Test of Activities of Daily Living; PP=per protocol; NOSGER=Nurses’ Observation Scale of Geriatric Patients.

^a^ Excluded due to a lack of variance: educational attainment (93% with no more than elementary school education), excluded due to multicollinearity (r≥0.50 with ADAS-Cog at baseline and/or E-ADL test at baseline): care level (0.76 with ADAS-Cog; 0.56 with the E-ADL test), Charlson comorbidity index (0.52 with ADAS-Cog); MMSE (0.71 with ADAS-Cog; 0.56 with the E-ADL test).

^b^ ADAS-Cog at baseline if ADAS-Cog 12-month follow-up is dependent variable; E-ADL test at baseline if E-ADL test 12-month follow-up is dependent variable.

^c^ Sedating (<0) or stimulating (>0) effect or side effect of the prescribed medications.

^d^ NOSGER-mood subscale, which consists of 5 depression items rated on a scale of 1 (always) to 5 (never). The score ranges from 5 (no disturbance) to 25 (maximum possible degree of disturbance).

^e^ Sum score of all non-MAKS activities regularly offered by the nursing homes and in which patients participated on a voluntary basis. The scores were recorded by nursing staff each week throughout the observation period. Examples of such activities are singing groups or physical exercises to reduce the risk of falling. Each activity in which a patient participated was rated with one point.

^f^ Use of acetylcholinesterase (AChE) inhibitors or Memantine.

^g^ The care level describes the extent to which nursing care is needed: none (no needs), 1 (moderate needs), 2 (high needs), and 3 (very high needs). This scale is used in Germany to establish eligibility for nursing care benefits. Put simply, individuals who need assistance with activities of everyday living and household chores for a daily average of at least 1.5 hours are assigned to level 1, those who need at least 3 hours of assistance to level 2, and those who need 5 or more hours of assistance to level 3. The classification is conducted by an organisation formed by the long-term care insurance funds, which are statutory entities that administer the system of long-term care insurance in Germany.

**APPENDIX 1 b. Multiple regression analysis with ADAS-Cog and E-ADL test as dependant variable (PP analysis, n=61)** **and with “education” as additional independent variable**

|  | **ADAS-Cog (12-month follow-up)** | | | **E-ADL test (12-month follow-up)** | | |
| --- | --- | --- | --- | --- | --- | --- |
| **Independent variable^a^** | Unstandardised β (95% CI) | *t* | P value | Unstandardised β (95% CI) | *t* | P value |
| **Score^b^ at baseline** | 0.80 (0.57-1.04) | 6.91 | <**0.001** | 0.81 (0.55-1.07) | 6.36 | **<0.001** |
| **Group (control=0 vs. MAKS=1)** | -7.58 (-13.88—1.29) | -2.42 | **0.019** | 3.45 (0.63-6.28) | 2.45 | **0.018** |
| **Age** | 0.09 (-0.51-0.69) | 0.30 | 0.77 | 0.10 (-0.17-0.37) | 0.76 | 0.45 |
| **Gender** | 1.37 (-6.58-9.32) | 0.35 | 0.73 | 0.71 (-2.82-4.24) | 0.41 | 0.69 |
| **Medication score^c^** | -0.36 (-2.13-1.40) | -0.42 | 0.68 | -0.03 (-0.81-0.76) | -0.07 | 0.95 |
| **NOSGER, mood^d^** | 0.81 (-0.21-1.84) | 1.59 | 0.12 | -0.14 (-0.59-0.31) | -0.64 | 0.53 |
| **Participation score^e^** | -0.07 (-0.14-0.00) | -2.01 | **0.05** | 0.01 (-0.02-0.04) | 0.54 | 0.59 |
| **Use of antidementia medication^f^** | -5.23 (-15.79-5.33) | -0.99 | 0.33 | 0.36 (-4.30-5.01) | 0.16 | 0.88 |
| **education** | 4.67 (-4.33-13.67) | 1.04 | 0.30 | -2.85 (-6.80-1.10) | -1.54 | 0.15 |

Abbreviations: ADAS-Cog=Alzheimer’s Disease Assessment Scale, subscale cognition; E-ADL test=Erlangen Test of Activities of Daily Living; PP=per protocol; NOSGER=Nurses’ Observation Scale of Geriatric Patients.

^a^ Excluded due to a lack of variance: educational attainment (93% with no more than elementary school education), excluded due to multicollinearity (r≥0.50 with ADAS-Cog at baseline and/or E-ADL test at baseline): care level (0.76 with ADAS-Cog; 0.56 with the E-ADL test), Charlson comorbidity index (0.52 with ADAS-Cog); MMSE (0.71 with ADAS-Cog; 0.56 with the E-ADL test).

^b^ ADAS-Cog at baseline if ADAS-Cog 12-month follow-up is dependent variable; E-ADL test at baseline if E-ADL test 12-month follow-up is dependent variable.

^c^ Sedating (<0) or stimulating (>0) effect or side effect of the prescribed medications.

^d^ NOSGER-mood subscale, which consists of 5 depression items rated on a scale of 1 (always) to 5 (never). The score ranges from 5 (no disturbance) to 25 (maximum possible degree of disturbance).

^e^ Sum score of all non-MAKS activities regularly offered by the nursing homes and in which patients participated on a voluntary basis. The scores were recorded by nursing staff each week throughout the observation period. Examples of such activities are singing groups or physical exercises to reduce the risk of falling. Each activity in which a patient participated was rated with one point.

^f^ Use of acetylcholinesterase (AChE) inhibitors or Memantine.

**APPENDIX 2 Overview of the intervention components with time-frame and 10 examples per component^a^**

Part 1 January to June

| **Time frame** | **Component** | **Example 1**  **January** | **Example 2**  **March** | **Example 3**  **April** | **Example 4**  **May** | **Example 5**  **June** |
| --- | --- | --- | --- | --- | --- | --- |
| 10 minutes | Introduction with spiritual elements | Epiphany (3 Wise Men) | The senses - vision | Easter | Motto “Friendship” | The senses - taste |
| 30 minutes | (Psycho-)motor exercises | Warm-up phase: Relaxing exercises  Main part:  Table football^b^ | Warm-up phase: Relaxing exercises  Main part:  clapper and balloons^c^ | Warm-up phase: Relaxing exercises  Main part  Motto “Circus” (Hoops, wooden stick) ^d^ | Warm-up phase: Relaxing exercises  Main part  Movement with the magic rope^e^ | Warm-up phase: Relaxing exercises  Main part  Croquet^f^ |
| 10 minutes | Break |  |  |  |  |  |
| 30 minutes | Cognitive activation | Warm-up phase  Finger gymnastics  Main part:  Combining numbers^l^ | Warm-up phase:  Pencil gymnastics  Main part:  Photo puzzle^m^ | Warm-up phase:  Finger gymnastics  Main part:  Counting exercise^n^ | Warm-up phase:  Pencil gymnastics  Main part:  ABC-exercise^o^ | Warm-up phase:  Finger gymnastics  Main part:  Combining sentences and words^p^ |
| 40 minutes | Activities of daily living | Making hot chocolate | Create a domino game | Weaving | Working in a garden bed | Making fruit salad |

Part 2 July to December

| **Time frame** | **Component** | **Example 6**  **July** | **Example 7**  **August** | **Example 8**  **October** | **Example 9**  **November** | **Example 10**  **December** |
| --- | --- | --- | --- | --- | --- | --- |
| 10 minutes | Introduction with spiritual elements | Motto “Mein Name” | Sources of refreshment | Life – Joy of living | All Saints | Symbols of the Christmas festival |
| 30 minutes | (Psycho-)motor exercises | Warm-up phase:  Relaxing exercises  Main part:  Exercise with the pool noodles^g^ | Warm-up phase: Relaxing exercises  Main part:  Foot gymnastics in the pool^h^ | Warm-up phase: Relaxing exercises  Main part:  Movement to song: “La Paloma” with real castagnettes^i^ | Warm-up phase: Relaxing exercises  Main part:  Indoor boccia^j^ | Warm-up phase: Relaxing exercises  Main part:  blanket and Ball^k^ |
| 10 minutes | Break |  |  |  |  |  |
| 30 minutes | Cognitive activation | Warm-up phase:  Pencil gymnastics  Main part:  Dice game^q^ | Warm-up phase:  Finger gymnastics  Main part:  Copying a figure^r^ | Warm-up phase:  Pencil gymnastics  Main part:  Counting symbol pairs^s^ | Warm-up phase:  Finger gymnastics  Main part:  Letter-counting text^t^ | Warm-up phase:  Pencil gymnastics  Main part:  What doesn’t fit in? ^u^ |
| 40 minutes | Activities of daily living | Preparing fruit punch | Making icecream with an ice-cream maker | Preparing cheese-grape cocktail sticks | Building a birdhouse | Making stars out of wooden beads |

^a^Intervention is described in detail in: Eichenseer, B., Graessel, E. (Eds.). (2011). *Aktivierungstherapie für Menschen mit Demenz - motorisch - alltagspraktisch - kognitiv – spirituell [Activating Intervention for Dementia Patients – motor – activities of daily living – cognititive - spiritual]* München: Elsevier, pp.1-314

**Explanations of (Psycho-)motor exercises**

^b^Table football is played in pairs

^c^A balloon is moved from player to player using a clapper (stick with foam rubber balls at the ends)

^d^The wheel (a colored rubber ring) is turned forwards and backwards using a wooden stick

^e^The participants exercise to the rhythm of music, bound together by a knotted gymnastic band

^f^The participants take turns aiming with the croquet mallet at the wickets stuck in the lawn

^g^Gymnastic exercises using the “pool noodles” (Flexible foam rubber sticks)

^h^Foot gymnastics are performed outdoors in an inflatable pool filled with water

^i^The participants accompany the song “La Paloma” rhythmically using real wooden castagnettes

^j^Boccia (leather balls) is played indoors on a smooth floor

^k^A ball is moved in all directions using a large blanket

**Explanations of Cognitive activation**

^l^Numbers are connected in rising sequence

^m^Spatial assignment of 4 parts of a picture

^n^Counting objects (like Easter eggs). The number of degrees of difficulty (1-3) depends on the number of objects

^o^Word association (e.g. what words or symbols do you think of in association with the color blue/red/green/yellow)

^p^Appropriate words and parts of sentences must be brought together

^q^Dice are thrown by the participants one after another. Each participant enters the number he thrown in a box on the exercise sheet. The points are counted at the end.

^r^a drawing of a simple figure is given and is to be copied.

^s^Symbol pairs with the same color are counted

^t^In a text (such as a poem), the letters marked with red (like. s,n,a or e) are to be counted and the number entered in a box.

^u^One of 4 objects which don’t belong to a main category (like household objects) must be recognized (such as tools)

**APPENDIX 3 Example for an exercise of cognitive activation with 3 different levels of difficulty**

Level 1 (easy) to 3 (difficult): Please count the laughing faces as fast as possible


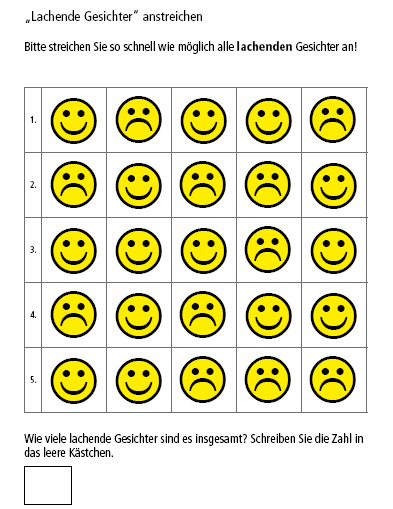


**APPENDIX 4 Non-MAKS Activities offered by the nursing homes**

| **Groups with an emphasis on  motor activities^a^** | **Groups with an emphasis on activities of daily living^b^** | **Groups with an emphasis on cognition^c^** | **Spiritual offers^d^** |
| --- | --- | --- | --- |
| physical exercises to reduce risk of falling | painting, doing handicrafts | reading and talking about newspaper articles | different religious groups |
| dancing in the group | house-keeping activities | choir | church services |
| gymnastics | cooking group | watching movies together | Bible study group |
| sitting dance |  | singing | pastoral care |
| sports |  | slide-shows |  |
| training of physical balance |  | memory training |  |
| aquarobics/ hydrotherapy |  | reading and listening to (fairy) tales, poetry and talking about them |  |
| Singing and Moving |  | playing parlor games (e.g. ludo, memory games) |  |
| bowling |  | various moderated groups for talking about special subjects |  |

^a^ 17.58 % of all non-MAKS-activities attended by all study participants

^b^  2.15 % of all non-MAKS-activities attended by all study participants

^a^ 53.52 % of all non-MAKS-activities attended by all study participants

^a^ 26.76 % of all non-MAKS-activities attended by all study participants
